# TiO_2_ supported pallidum-bipyridyl complex as an efficient catalyst for Suzuki–Miyaura reaction in aqueous-ethanol

**DOI:** 10.1038/s41598-024-57534-9

**Published:** 2024-03-27

**Authors:** Upendar Reddy Gandra, Pogula Sreekanth Reddy, Amatus Salam, Surya Prakash Gajagouni, Akram Alfantazi, M. Infas H. Mohideen

**Affiliations:** 1 Department of Chemistry, Khalifa University, P.O. Box 127788, Abu Dhabi, United Arab Emirates; 2 Center for Global Infectious Disease Research, Seattle Children’s Research Institute, Seattle, WA 98109 USA; 3 Department of Mechanical Engineering, Khalifa University, P.O. Box 127788, Abu Dhabi, United Arab Emirates; 4 Department of Chemical Engineering, Khalifa University, P.O. Box 127788, Abu Dhabi, United Arab Emirates; 5 Center for Catalysis and Separations, Khalifa University of Science and Technology, P.O. Box 127788, Abu Dhabi, United Arab Emirates

**Keywords:** Chemistry, Materials science, Nanoscience and technology

## Abstract

Owing to their improved catalytic stability and ability to undergo repeated cycles, solid-supported catalysts show great potential for various catalytic reactions. In this study, we synthesized a catalyst comprising a palladium-2,2-bipyridine complex supported on TiO_2_ nanoparticles (TiO_2_@BDP-PdCl_2_) fully characterised and investigated its efficacy in Suzuki–Miyaura cross coupling reactions involving phenyl boronic acid with various aryl halides under mild reaction conditions. The 2,2′- bipyridine (bp) has shown excellent complexation properties for Pd (II) and it could be easily anchored onto functionalized TiO_2_ support by the bridging carboxylate ions. The composition and structure of the as-prepared catalyst was characterized by powder X-ray diffraction (PXRD), scanning electron microscopy (SEM), Transmission Electron Microscope (TEM), X-ray photoelectron spectroscopy (XPS), and UV–Vis spectroscopy. The catalyst easily demonstrated separability, enhancing its practicality in catalytic processes. Subsequent utilization showed a consistent activity level, suggesting the stabilization of the aggregated catalyst species. This research sheds light on the importance of catalyst stability and maintenance during consecutive reaction cycles.

## Introduction

The design of robust catalysts is important for catalytic systems in the view of organic chemistry. Transitional metals and their complexes play a very important role during the formation of carbon–carbon bond. Among the transition metals, palladium-based Suzuki–Miyaura cross-coupling reaction of aryl halides with arylboronic acids has become a convenient synthetic method in organic chemistry^1–5^. Palladium (Pd) nanoparticle works well in catalytic coupling reactions to some extent, however catalysts suffer from obvious aggregation after several cycles and considerable metal leaching which is responsible for the decrease or loss of their intrinsic catalytic activities^6–11^. This is probably due to the lack of sufficient binding sites on the support materials, where only weak non-covalent interactions exist between the support materials and the metal nanoparticles^12,13^. Given the cost of palladium and its toxic nature, the separation, recovery, and reutilization of palladium catalysts are crucial. This underscores the heightened demand for non-aggregated metal nanoparticles in coupling reactions, aiming to improve the overall efficiency of the catalyst. The aggregation of Pd nanoparticles can be overcome or minimized by immobilizing them on some solid supports, such as carbon, metal oxides, and polymers which could generate strong covalent interaction with palladium species and stabilize the catalyst^8,9,14–19^. In this context, the distinctive features of heterogeneous palladium catalysts include their heightened catalytic sites, exceptional selectivity, ability to control catalyst chemo-, regio-, and enantioselectivities, ease of optimizing catalytic systems, and enhanced yields, making them widely employed in Suzuki coupling reactions^16,20,21^. The challenge at hand pertains to the design and synthesis of expensive ligands essential for constructing heterogeneous catalysts, particularly when anchoring them onto solid supports through covalent attachments^22,23^. As a result, many cost-effective materials such as silica, carbon, zeolite, cellulose, and chitosan were explored as alternative support options^14,18,24–29^. However, the exploration of a Pd catalyst supported by TiO_2_ nanoparticles in Suzuki reactions is limited in the current literature. Despite the numerous advantages it offers, including heightened catalytic activity, tailored reactivity, improved selectivity, as well as enhanced stability, durability, and recyclability, this approach remains relatively under explored^22,30^. Researchers has demonstrated that TiO_2_ significantly enhances catalyst performance, affording the ability to modulate catalytic activities for diverse reactions, spanning dehydrogenation^31^, hydrodesulphurization^32^, water gas shift^33^, and thermal catalytic decomposition^34,35^. The interactions between catalytic particles and mesoporous TiO_2_ play a pivotal role, profoundly influencing catalytic activity, stability, and selectivity in heterogeneous metal catalysts^16^. However, it's important to acknowledge that these electrostatic interactions may entail certain drawbacks, including weak bonding, limited stability under harsh conditions, diminishing catalytic activity over time, potential metal loss during catalyst preparation, and heightened sensitivity to reaction conditions, among others^36^. These considerations necessitate careful attention when designing and employing such catalyst systems. By keeping these limitations, we were interested to design chemical modification of TiO_2_ nanoparticles for stable supported heterogeneous catalytical applications, in particular under organic solution conditions, in which the surface of TiO_2_ nanoparticles needs the modification by organic modifiers. Carboxylic acids are often used as such modifiers, with a coordination of carboxylic groups (-COOH) to surface Ti atoms^37^. In this paper, for the first time we demonstrate a new and convenient solvothermal approach to chemically postmodify TiO_2_ nanoparticles with di-carboxylic acids derived from chelated Pd bipyridyl complexes. The resulting material demonstrates enhanced catalytic performance in Suzuki Coupling Reactions, showcasing its potential in various catalytic applications. This approach opens new avenues for the design and development of advanced heterogeneous catalysts for organic synthesis. To the best of knowledge, anchoring of palladium-2,2-bipyridine complex on TiO_2_ surface not reported yet in literature for catalytic applications.

## Experimental

### Materials & characterization

All commercially available solvents, unless otherwise mentioned, were used without any other purification. All chemicals were procured from Aldrich and Across chemical companies and used as-received without any further purification. Glassware was dried in an oven prior to use. Reactions were monitored by thin-layer chromatography (TLC) with Merck silica gel 60 F254 plates_._ Column chromatography was performed on silica gel 100–200 mesh from SDFCL. ^1^H and ^13^C NMR spectra were recorded on a Bruker DRX 500 MHz spectrometers using TMS (^1^H) as an external standard. Chemical shifts (*δ*) are reported in ppm. The coupling constants *J* are given in Hz. Fourier transform infrared (FTIR, VERTEX 70, Bruker) spectra were recorded to analyse the functional groups present in all the samples. X-ray diffraction measurements were performed on Rigaku Smart Lab II with Cu K_alpha (λ = 1.5405 Å) radiation source operating at 40 kV and 40 mA. Scanning electron microscopy (SEM, JEOL JSM-7610FFEG-SEM) were employed to observe the morphology. Thermogravimetric analysis (TGA, TA-Q50) was employed to determine the thermal stability of catalyst under N_2_ at a heating rate of 10 °C min^−1^ in a temperature range of 25–600 °C.

### Preparation of TiO_2_ nanoparticles^37^:

The mixture of 2-propanol (2.5 mL, AR) and Titanium isopropoxide (6 mL) was added dropwise over 10 min into 0.1 M nitric acid solution (30 mL) under vigorous stirring at room temperature, and then the reaction mixture was heated to 80 °C and stirred vigorously for 10 h to achieve peptization. A Buchner funnel was used to remove the non-peptized agglomerates, and the filtrate was transferred into a Teflon-lined stainless-steel autoclave with a capacity of 100 mL. After heating at 200 °C for 12 h, the autoclave was allowed to cool to room temperature. The nanoparticles were isolated by centrifugation and washed with ethanol. The crude TiO_2_ NPs were then washed with ethanol three times to remove the unreacted precursor. The TiO_2_ NPs were dried in vacuo and stored in a sealed container at room temperature for further usage.

### Synthesis of BDP-PdCl_2_^38^:

In the dark and under nitrogen atmosphere, PdCl_2_ (80 mg, 0.45 mmol) and KCl (67 mg, 0.90 mmol) were dissolved in a 10 mL solution consisting of Methanol: Water in a 9:1 ratio and refluxed it for 3 h. To this reaction mixture, H_2_BDP (109 mg, 0.45 mmol) dissolved in 2 mL of ethanol was added. The reaction mixture was left to reflux for 12 h and filtered off. The moist product was subsequently suspended in water, which was prepared with 6–7 drops of 6 M HCl, and heated for an hour or more until it reduced to 1/3 of its initial volume. Afterward, the mixture was cooled in an ice bath and filtered once more. It was then washed with cold distilled water to retrieve light brownish coloured compound (80% yield). IR (KBr, cm^−1^): 3448 (s), 1677 (s), 1662 (w), 1607 (m), 1542(m), 1407(s), 1386(s), 1289(w), 1268(w), 1131(w), 786(m).

### Synthesis of TiO_2_@BDP-PdCl_2_^39^:

BDP-PdCl_2_ was dissolved into acetonitrile (CH_3_CN) solvent (3 mg/mL) to prepare a stock solution. Then, TiO_2_ NPs (1.5 mg/mL in acetonitrile) were mixed with the BDP-PdCl_2_ solution (3 mg/mL in acetonitrile) in a 1:1 volume ratio and stirred for 24 h. The mixture was then centrifuged to obtain TiO_2_@BDP-PdCl_2_ NPs. Product was purified by centrifugation and washed with methanol to remove unbound BDP-PdCl_2_. This step was repeated three times to obtain the final TiO_2_@BDP-PdCl_2_ product. The obtained TiO_2_@BDP-PdCl_2_ NPs were then dried in vacuo and stored at room temperature.

### General reaction conditions for Suzuki coupling reactions

In a 25 mL reaction vial were placed aryl halide (1 mmol), phenylboronic acid (1.2 mmol), K_2_CO_3_ (1.2 mmol), and TiO_2_@BDP-PdCl_2_ (10 mg) in 1:1 ethanol/water (5 mL) and the resulting mixture was stirred at 80 ℃ for 3 h. The reaction was monitored by TLC and after the reaction, the mixture was extracted with ethyl acetate. The organic layer was dried with sodium sulphate, filtered, and concentrated in *vacuo*. The residue was then purified by column chromatography over silica gel (100 − 200 mesh size) with petroleum ether-ethyl acetate as the eluent. The products were confirmed with ^1^H and ^13^C NMR spectroscopic analysis.

### General procedure for catalyst recovery

Iodobenzene (5.0 mmol), phenylboronic acid (6.0 mmol), K_2_CO_3_ (6.0 mmol), TiO_2_@BDP-PdCl_2_ (10 mg), and ethanol/H_2_O (25 mL) were placed in a 100 ml reaction flask and stirred at 80 ℃ for 6 h. After the reaction, ethyl acetate, ethanol, and deionized water were added successively for centrifugal cleaning, the catalyst and the product were separated by using centrifugation for 15 min at 10,000 rpm. The residual catalysts were then dried at room temperature under vacuum before being used again for the next reaction.

## Results and discussions

**Scheme 1:**
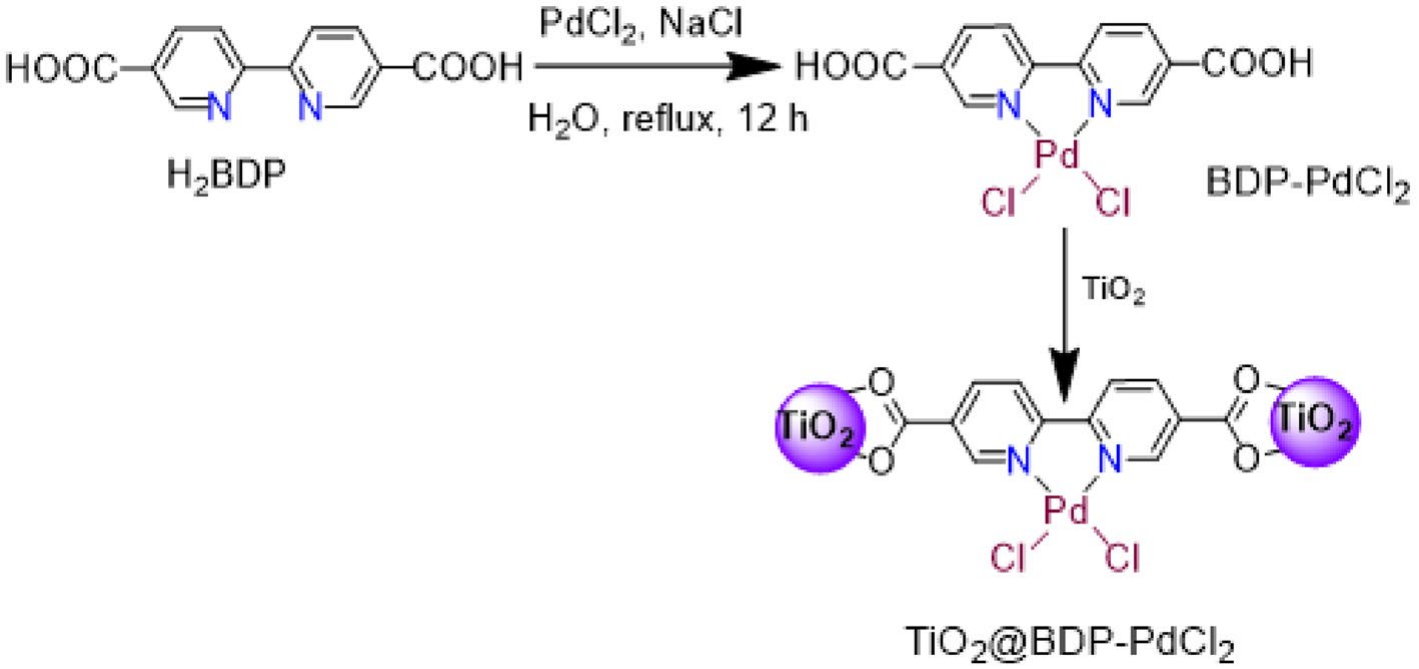
Reaction scheme adopted for palladium complex onto bipyridyl-functionalized TiO_2_ through coordinative attachment.

In this study, H_2_BDP was employed for coordination with PdCl_2_, resulting in the formation of BDP-PdCl_2_. Subsequently, the BDP-PdCl_2_ complex was firmly attached to the TiO_2_ particle surface shown in Scheme 1. Analytical and spectroscopic assessments substantiated the successful synthesis and high purity of both the BDP-PdCl_2_ ligand and TiO_2_@BDP-PdCl_2_ composite.

**Figure 1:**
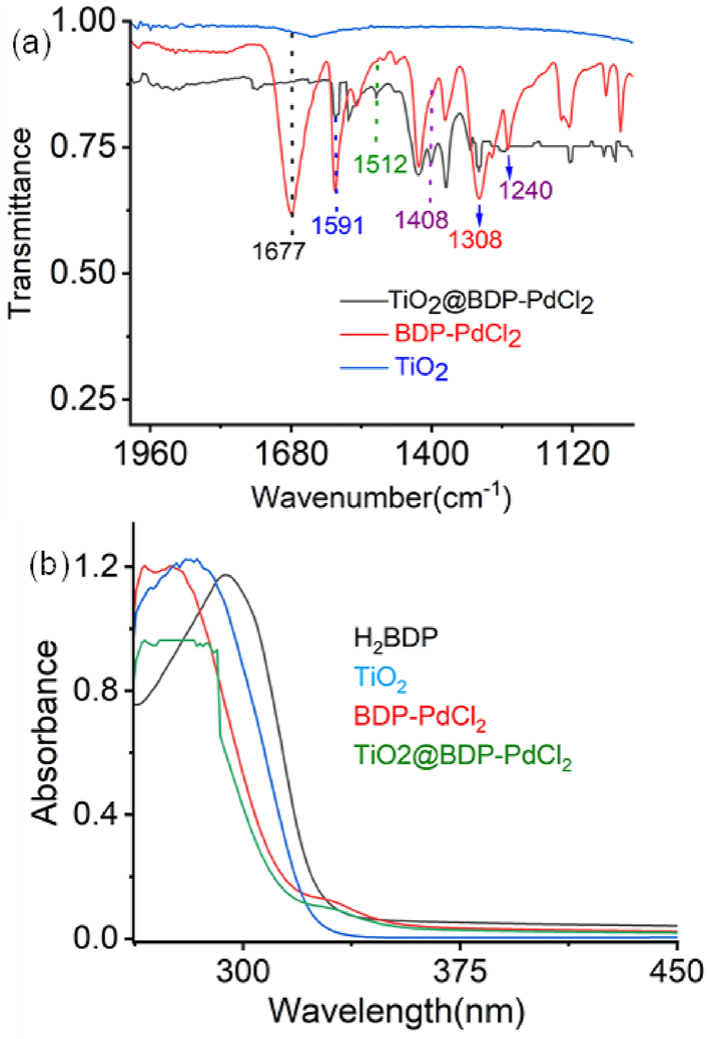
(**a**) FT-IR spectra of only TiO_2_ nanoparticles (blue line), only BDP-PdCl_2_ (red line) and TiO_2_ nanoparticles modified with BDP-PdCl_2_ (black line) via solvothermal modification. (**b**) Absorption spectra of H_2_BDP before and after Pd complexation. Absorption spectra of TiO_2_ before and after BDP-PdCl_2_ anchoring.

FT-IR spectra of the TiO_2_ nanoparticles modified with BDP-PdCl_2_ are shown in Fig. 1a. In the spectrum of BDP-PdCl_2_ the bands are attributed to the stretching and bending coupled C–OH vibrations (ν_C-OH_, at 1308–1275 cm^−1^), the in-plane bending of O–H (*δ*_O-H_, at 1408 cm^−1^), and the stretching band of the carboxylic acid (*ν*_C=O_, at 1677 cm^−1^), while the band at 1591 cm^−1^ is due to the C=C stretching vibration of phenyl ring (ν C = C). TiO_2_@BDP-PdCl_2_ showed the ν_C=C_ band at 1591 cm^−1^ as well as the carboxylate anion (COO^−^) asymmetric (ν_as_, at 1512 cm^−1^) and symmetric (ν_s_, at 1408 cm^−1^) stretching bands due to the splitting of carboxylate groups complexed with Ti surface centres. Notably, the absence of the ν_C=O_ stretching vibration of carboxylic acid at 1677 cm^−1^ in TiO_2_@BDP-PdCl_2_ further supports the formed complex.

Moreover, to delve into the intricacies of Pd complexation, encompassing the stages pre- and post-complexation, as well as the subsequent anchoring of BDP-PdCl_2_ onto the TiO_2_ surface, we executed an extensive array of UV–Vis studies (Fig. 1b). H_2_BDP exhibited a conspicuously robust absorption band peaking at 295 nm, attributed to the characteristic bipyridyl ligand-cantered n–π*-based transitions. In contrast, BDP-PdCl_2_ manifested a relatively subdued and broader band at 333 nm, correlated with a spin-allowed dπ_Pd(II)-_π*_BDP_-based metal-to-ligand charge transfer (^I^MLCT) transition along with intense absorption band at 280 nm, accompanied by a blue shift (Δλ = 10 nm), providing confirmation of the formation of the Pd complexation^40–42^. Furthermore, the absorption intensity of TiO_2_@BDP-PdCl_2_ was observed to be less pronounced and broader compared to BDP-PdCl_2_. The UV–Vis spectrum of TiO_2_@BDP-PdCl_2_ exhibited distinct broad absorption bands at 280 and 329 nm respectively, confirming the anchoring of BDP-PdCl_2_ onto the Ti surface. These absorption patterns align with the findings from the FT-IR.

**Figure 2:**
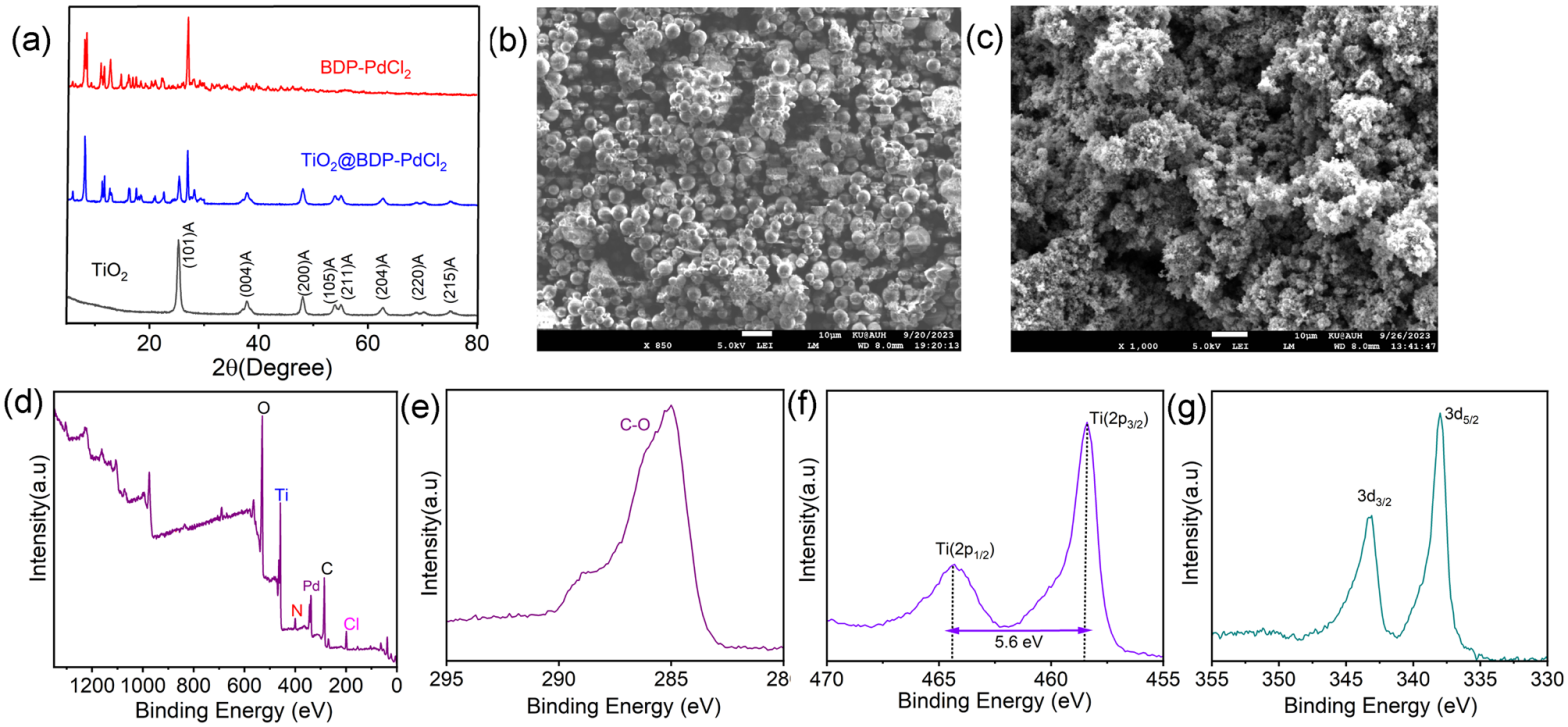
(**a**) X-ray diffraction (XRD) patterns of TiO_2_, BDP-PdCl_2_,TiO_2_@BDP-PdCl_2_. Scanning electron microscopy (SEM) images of (**b**) only TiO_2_, (**c**) TiO_2_@BDP-PdCl_2_. XPS spectra of TiO_2_@BDP-PdCl_2_ (**d**) wide, (**e**) C1s, (**f**) Ti 2p, and (**g**) Pd 3d.

In order to further characterize the crystalline composition, shape, and surface coverage after the solvothermal reaction, the samples were studied by XRD, SEM, XPS, and TGA measurements. The X-ray diffraction (XRD) pattern exhibits prominent and well-defined peaks, indicating the crystalline nature of the samples (Fig. 2a). Initially, the presence of characteristic peaks at 2θ values of 25.3°, 37.9°, 48.0°, 54.7°, 62.9°, 70.0°, and 75.3° confirms the anatase phase of TiO_2_, corresponding to the (101), (004), (200), (211), (204), (220), and (215) planes, respectively. Furthermore, in the XRD pattern of TiO_2_@BDP-PdCl_2_, we observed both newly emerging peaks and characteristic anatase peaks of TiO_2_, providing definitive evidence for the formation of TiO_2_@BDP-PdCl_2_. Post-modification of TiO_2_, as a result slight change in the morphology along with the porous surface was observed in the SEM micrographs (Fig. 2b*, *c). TG − differential TG (DTG) diagrams of TiO_2_@BDP-PdCl_2_ and TiO_2_ are shown in Figure S5. Both exhibit a nearly 7% weight loss at around 100 °C, attributed to the desorption of surface-adsorbed water molecules. Notably, TiO_2_ demonstrates superior thermal stability owing to its high degree of crystallinity. TiO_2_@BDP-PdCl_2_ exhibits a 60% thermal degradation at 400 °C, indicating commendable thermal stability of the catalyst below this temperature threshold. X-ray photoelectron spectroscopy (XPS) was employed to analyze the chemical and electronic states of carbon (C), palladium (Pd), and titanium (Ti) (Fig. 2d, e). Examination of the XPS spectrum of the 3p orbitals of the Ti core revealed two distinct peaks at binding energies (BE) of 458.41 eV and 464.32 eV. These peaks correspond to the Ti (2p_3/2_) and Ti (2p_1/2_) core levels of Ti^4+^ cations, indicating the absence of Ti^3+^. Moreover, the substantial energy gap of 5.91 eV between the Ti (2p_3/2_) and Ti (2p_1/2_) peaks, along with their area ratio of 3.45, signifies the robust bonding between titanium (Ti) and oxygen (O) atoms of anatase form of TiO_2_ nanoparticles (Fig. 2f)^43^. In the case of the Pd core, the XPS spectrum of the 3d orbitals exhibited peaks at approximately 338.01 eV and 343.17 eV for Pd 3d_5/2_ and Pd 3d_3/2_, respectively. Both Pd peaks are attributed to the Pd (II) state (Fig. 2g)^44^. The mass fraction of Ti on TiO_2_@BDP-PdCl_2_ reached 9.96%, which is similar to the amount of the metal precursor.

### Application of TiO_2_@BDP-PdCl_2_ for the Suzuki- Miyaura cross coupling reaction

After characterizing the structure and properties of TiO_2_@BDP-PdCl_2_ catalyst was then examined for the carbon − carbon (C–C) bond formation reaction *i.e*., Suzuki–Miyaura cross coupling reaction. The C–C cross coupling of iodobenzene and phenylboronic acid were utilized as a model for the optimization of reaction conditions (Table 1). The effect of different reaction factors such as solvent, base, temperature, reaction time and catalyst amount were evaluated. In the initial phase of our investigation, we scrutinized the influence of various solvents (Table 1, entries 1 − 8). The use of water (H_2_O) led to the formation of the desired product, biphenyl, albeit with a lower yield of 34% after 6 h (Table 1, entry 1). In contrast, the use of a nonpolar solvent like toluene resulted in a substantial yield of biphenyl (Table 1, entry 2). Subsequently, a series of polar solvents including tetrahydrofuran (THF), dioxane, and acetonitrile were evaluated, and they exhibited efficiency by providing moderate to good yields (Table 1, entries 3 − 5). The utilization of a polar protic solvent such as ethanol was also explored, yielding a satisfactory product (Table 1, entry 6). Following this, we investigated the use of polar aprotic solvents such as DMF and propylene carbonate [7c], which produced biphenyl with yields of 74% and 45% respectively (Table 1, entry 7 & 8). Inspired by the previous reports^11,25,45^, the effect of a binary mixture of solvents containing ethanol/water (1:1) was investigated and delivers an excellent yield of biphenyl in 95% yield for 3 h (Table 1, entry 9). Next, we explored the impact of different bases on the reaction system. The employment of inorganic bases like Cs_2_CO_3_ and Na_2_CO_3_ yielded favourable biphenyl yields (Table 1, entries 9 − 11). Conversely, the utilization of organic bases like Et_3_N resulted in a 59% yield after 3 h (Table 1, entry 12). Following this, we assessed the influence of temperature on the reaction. It was observed that reducing the reaction temperature to 60 °C and 40 °C led to a decrease in biphenyl yield (Table 1, entries 14 − 16). Subsequently, we conducted the reaction while varying the catalyst quantity. It is noteworthy that augmenting the catalyst quantity from 10 to 20 mg yielded no discernible enhancements (Table 1, entry 15). Conversely, decreasing the catalyst amount from 10 to 5 mg resulted in a slight reduction in yield (Table 1, entry 16).

**Table 1:** Optimization of Suzuki–Miyaura cross coupling reaction between iodobenzene and phenylboronic acid catalysed by TiO_2_@BDP-PdCl_2_.


Entry	Solvent	Base	Temp (℃)	Time (h)	Yield (%)^a^
1	H_2_O	K_2_CO_3_	80	6	34
2	Toluene	K_2_CO_3_	80	4	73
3	THF	K_2_CO_3_	80	4	71
4	Dioxane	K_2_CO_3_	80	3	78
5	CH_3_CN	K_2_CO_3_	80	4	65
6	EtOH	K_2_CO_3_	80	3	83
7	DMF	K_2_CO_3_	80	3	74
8	Propylene carbonate	K_2_CO_3_	80	6	45
9	EtOH/H_2_O	K_2_CO_3_	80	3	95
10	EtOH/H_2_O	Cs_2_CO_3_	80	3	93
11	EtOH/H_2_O	Na_2_CO_3_	80	3	90
12	EtOH/H_2_O	Et_3_N	80	3	59
13	EtOH/H_2_O	K_2_CO_3_	60	3	89
14	EtOH/H_2_O	K_2_CO_3_	40	3	81
15^b^	EtOH/H_2_O	K_2_CO_3_	80	3	95
16^c^	EtOH/H_2_O	K_2_CO_3_	80	3	85

Reaction conditions: Iodobenzene (1.0 mmol), phenylboronic acid (1.2 mmol), base (1.2 mmol), TiO_2_@BDP-PdCl_2_ (10 mg), and solvent (5 mL, 1:1 EtOH/H_2_O). ^a^Isolated yield after column purification, all reactions monitored by TLC. ^b^20 mg of TiO_2_@BDP- PdCl_2_. ^c^5 mg of TiO_2_@BDP-PdCl_2_.

We have further expanded the catalytic potential of TiO_2_@BDP-PdCl_2_ to encompass a broader array of biaryl substrates (Table 2) using carefully optimized reaction conditions. The reaction of iodobenzene and bromobenzene with phenylboronic acid yielded biphenyl with exceptional efficiency (Table 2, entries 1–2), while chlorobenzene resulted in notably lower yields (Table 2, entry 3). Subsequently, we conducted reactions between phenylboronic acid and various aryl halides possessing both electron-donating (-OCH_3_) and electron-withdrawing (-COCH_3_ & -CHO) groups. It was observed that bromine derivatives (Table 2, Entries 5 & 8) and iodine derivatives (Table 2, Entries 4, 7 & 10) exhibited excellent reactivity with phenylboronic acid, furnishing the corresponding coupled products with yields ranging from 90 to 94%. Conversely, the yield was significantly reduced when chloride derivatives were utilized as substrates (Table 2, Entry 6 & 9).

**Table 2:** TiO_2_@BDP-PdCl_2_ catalysed Suzuki − Miyaura coupling reaction of various aryl halides with phenylboronic acid.


Entry	R	X	Yield (%)^a^
1	H	I	95
2	H	Br	94
3	H	Cl	40
4	OCH_3_	I	94
5	OCH_3_	Br	92
6	OCH_3_	Cl	42
7	COCH_3_	I	92
8	COCH_3_	Br	91
9	COCH_3_	Cl	35
10	CHO	I	90

Reaction conditions: Aryl halide (1.0 mmol), phenylboronic acid (1.2 mmol), K_2_CO_3_ (1.2 mmol), TiO_2_@BDP-PdCl_2_ (10 mg) and 1:1 EtOH/H_2_O (5 mL). ^*a*^Isolated yield after column purification.

In pursuit of discerning the influence of TiO_2_ as a solid support, we conducted the C–C cross coupling of iodobenzene and phenylboronic acid exclusively employing BDP-PdCl_2_, under uniform conditions. The utilization of BDP-PdCl_2_ led to a notably low conversion rate of 72% and a yield of 66%. The outcome is in concordance with earlier documented findings^46^. This data substantiates the pivotal role of solid support in mitigating aggregation-induced activity during C–C bond formation. It is known that TiO_2_ exhibits both photocatalytic and catalytic properties. In order to assess the impact of TiO_2_ on C–C cross coupling reactions, we conducted a Suzuki reaction under nearly identical conditions. The reaction occurred in the presence of TiO_2_ but yielded unsatisfactory results, while no reaction occurred in the absence of Pd.

### Recyclability of the catalyst

**Figure 3:**
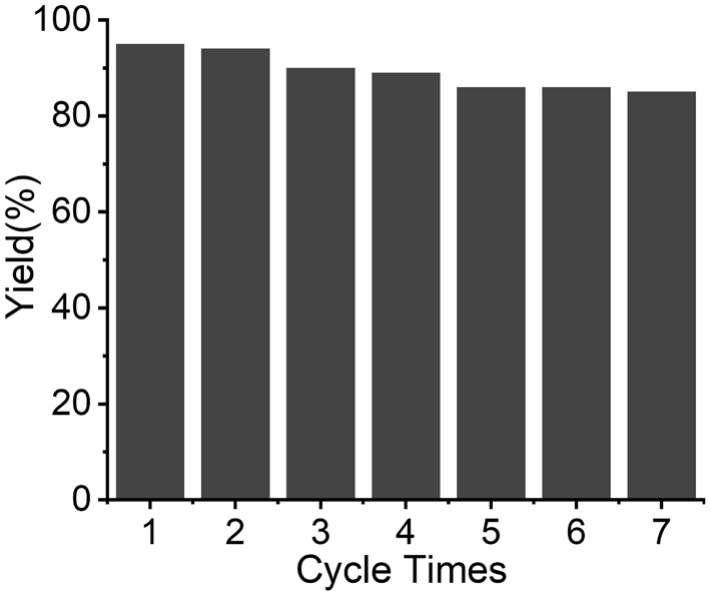
Recyclability of the TiO_2_@BDP-PdCl_2_ catalyst. Reaction conditions: Iodobenzene (1.0 mmol), phenylboronic acid (1.2 mmol), K_2_CO_3_ (2.0 mmol), ethanol/water (1:1), temperature (80 °C).

The developed TiO_2_@BDP-PdCl_2_ catalyst was then investigated for the recyclability by using a standard reaction condition for the Suzuki-Mayura cross coupling reaction of iodobenzene and phenylboronic acid. Remarkably, the TiO_2_@BDP-PdCl_2_ catalyst showed excellent recyclability, although the yield of the reaction decreased slightly after each run, it remained at 85% after seven cycles (Fig. 3). TEM images revealed that both the freshly prepared catalyst and the reused catalyst maintained their structural integrity, as evidenced in Fig SI 6. Additionally, XPS analysis provided further confirmation of the stability of TiO_2_@BDP-PdCl_2_, even after five catalytic cycles (Fig SI 7).

## Conclusions

In summary, TiO_2_ could be used as a solid support for Pd catalysis. TiO_2_ -supported Palladium-2,2-bipyridine complex was synthesized and fully characterized by various analytical techniques. The catalyst exhibits higher catalytic activity for the Suzuki–Miyaura coupling reaction. Moreover, TiO_2_@BDP-PdCl_2_ can be recycled and reused four times without a significant decrease in catalytic activity. The reactivity of the solid-supported catalyst remains high and enables the synthesis of various biphenyls from iodobenzene and phenylboronic acid derivatives. As of now, there is no existing documentation on the immobilization of the palladium-2,2-bipyridine complex on the TiO_2_ surface. We believe that a Pd catalyst supported by TiO_2_ has the potential to serve as a recyclable catalyst, demonstrating applicability in various C–C coupling reactions.

### Supplementary Information

Supplementary Information.

## Data Availability

All data generated or analysed during this study are included in this present article and available in supplementary information file.
