# Supplementary material for: TiO2 supported pallidum-bipyridyl complex as an efficient catalyst for Suzuki–Miyaura reaction in aqueous-ethanol
Source: Sci Rep. 2024 Mar 27;14:7323. doi: 10.1038/s41598-024-57534-9 (PMC10973498; doi:10.1038/s41598-024-57534-9)
Supplement: Supplementary file 1 — Supplementary Information. [file 41598_2024_57534_MOESM1_ESM.docx]

**Supporting Information**

**TiO_2_ Supported Pallidum-Bipyridyl Complex as an Efficient Catalyst for Suzuki–Miyaura Reaction in Aqueous-Ethanol**

Upendar Reddy Gandra,^a^ Pogula Sreekanth Reddy,^b^ Amatus Salam,^a^ Surya Prakash Gajagouni,^c^ Akram Alfantazi,^d^ M. Infas H. Mohideen^*a,e^

*^a^Department of Chemistry, Khalifa University, P.O. Box 127788, Abu Dhabi, United Arab Emirates; E-mail: mohamed.mohideen@ku.ac.ae*

*^b^Center for Global Infectious Disease Research, Seattle Children's Research Institute, Seattle, WA 98109, USA.*

*^c^Department of Mechanical Engineering, Khalifa University, Abu Dhabi. P.O. Box 127788, Abu Dhabi, United Arab Emirates.*

*^d^Department of Chemical Engineering, Khalifa University, Abu Dhabi. P.O. Box 127788, Abu Dhabi, United Arab Emirates.*

*^e^Center for Catalysis and Separations, Khalifa University of Science and Technology, Abu Dhabi P.O. Box 127788, United Arab Emirates.*

**The NMR data of products obtained *via* Suzuki-Miyaura coupling reaction.**

**1,1'-biphenyl** (Table 2, Entry 1, 2 & 3)**:** White solid. **^1^H-NMR (500 MHz, CDCl_3_)** δ 7.60- 7.58 (m, 4H), 7.45–7.42 (m, 4H), 7.36-7.32 (m, 2H).

**^13^C-NMR (125 MHz, CDCl_3_)** *δ*: 141.26, 128.75, 127.25, 127.17.

**4-Methoxy-1,1'-biphenyl** (Table 2, Entry 4, 5 & 6)**:** White solid. **^1^H NMR (500 MHz, CDCl_3_)** δ (7.56-7.52 m, 4H) 7.42 (t, *J* = 7.5 Hz, 2H), 7.31-7.28 (m, 1H, *)* 6.99 (d, *J* = 8. 5 Hz, 2H), 3.85 (s, 3H).

**^13^C NMR (125 MHz, CDCl_3_)** *δ* 159.16, 140.85, 133.80, 128.73, 128.17, 126.75, 126.67, 114.22 , 55.36.

**1-([1,1'-biphenyl]-4-yl)ethan-1-one** (Table 2, Entry 7, 8 & 9): White solid. **^1^H NMR (500 MHz, CDCl_3_)** δ 8.02 (d, 2H, *J* = 8.0 Hz), 7.67 (d, 2H, *J* = 8.0 Hz), 7.62-7.60 (m, 2H), 7.47 (t, 2H, *J* = 7.5 Hz), 7.40(t, 1H, *J* = 7.5 Hz), 2.62 (s, 3H).

**^13^C NMR (125 MHz, CDCl_3_):** δ 197.78, 145.79, 139.88, 135.88, 129.00, 128.95, 128.27, 127.3, 127.24, 26.69.

**[1,1'-biphenyl]-4-carbaldehyde** (Table 2, Entry 10): White solid. **^1^H NMR (500 MHz, CDCl_3_):** δ 10.02 (s, 1H). 7.93 (d, *J* = 7.5 Hz, 2H), 7.72 (d, 2H, *J* = 6.5 Hz), 7.61 (d, *J* = 7.5 Hz, 2H), 7.47 (t, *J* = 7.5 Hz, 2H), 7.41 (t, 1H, *J* = 7.0 Hz, 1H).

**^13^C NMR (125 MHz, CDCl_3_):** δ 191.93, 147.17, 139.71, 135.24, 130.29, 129.05, 128.52, 127.69, 127.39.

**2-(methoxymethyl)-1,1'-biphenyl:^1^H NMR (400 MHz, CDCl_3_)** δ 7.53 (dd, *J* = 7.2, 1.7 Hz, 1H), 7.44 – 7.31 (m, 7H), 7.30 – 7.26 (m, 1H), 4.33 (s, 2H), 3.32 (s, 3H). **^13^C NMR (100 MHz, CDCl_3_)** δ 141.9, 140.9, 135.4, 130.0, 129.3, 129.2, 128.1, 127.7, 127.5, 127.2, 72.5, 58.2

**4-nitro-1,1'-biphenyl:^1^H NMR (500 MHz, CDCl_3_)** δ 7.85 (dd, *J* = 8.1, 1.1 Hz, 1H), 7.62 (td, *J* = 7.6, 1.3 Hz, 1H), 7.48 (td, *J* = 7.9, 1.4 Hz, 1H), 7.46 – 7.40 (m, 4H), 7.32 (dt, *J* = 4.3, 2.4 Hz, 2H).**^13^C NMR (125 MHz, CDCl_3_)** δ 137.4, 136.4, 132.3, 131.9, 128.7, 128.3, 128.2, 127.9, 124.1.

**Figure S1.** ^1^H NMR & ^13^ C NMR spectra of 1,1'-biphenyl (Table 2, Entry 1, 2 & 3)


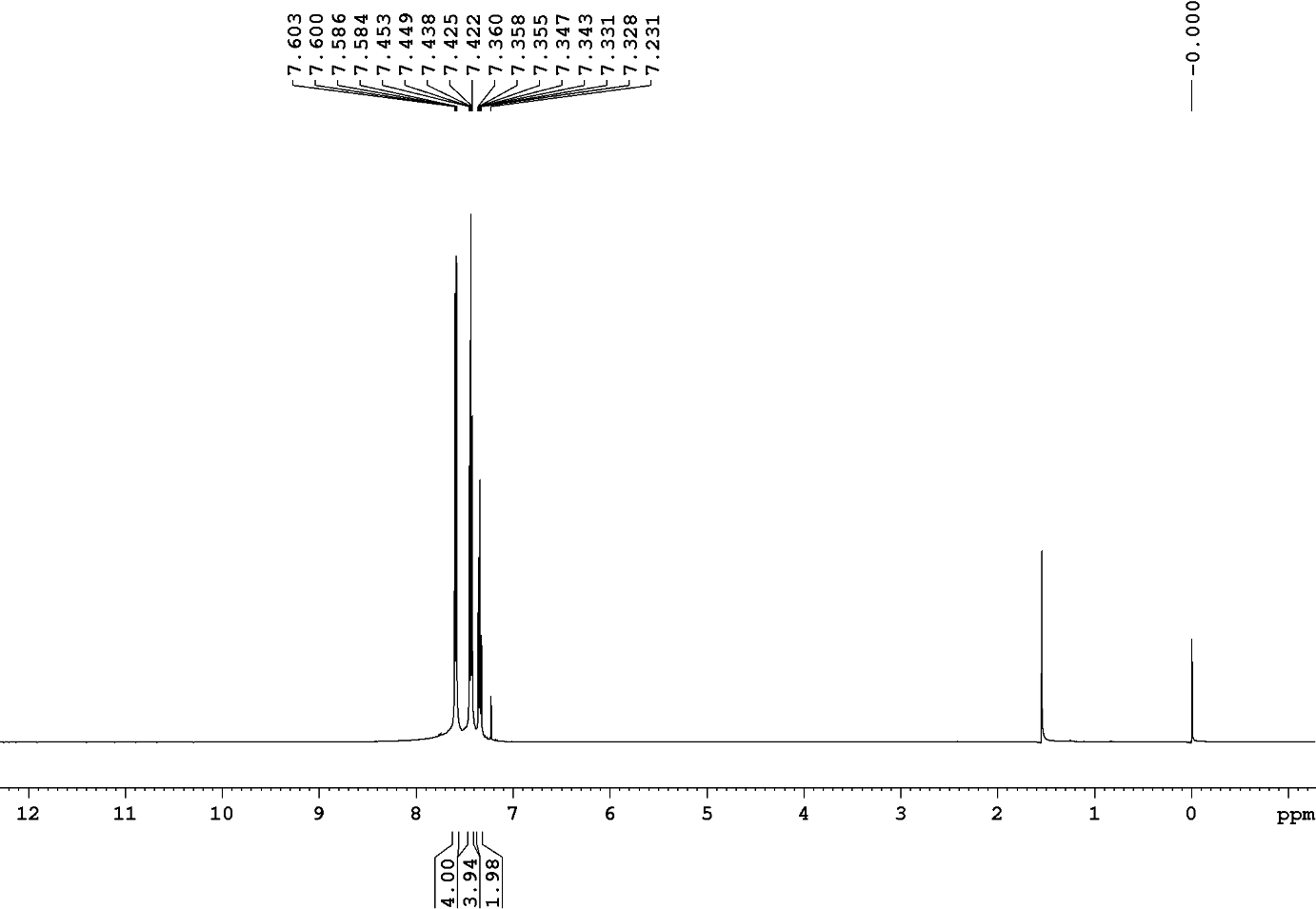

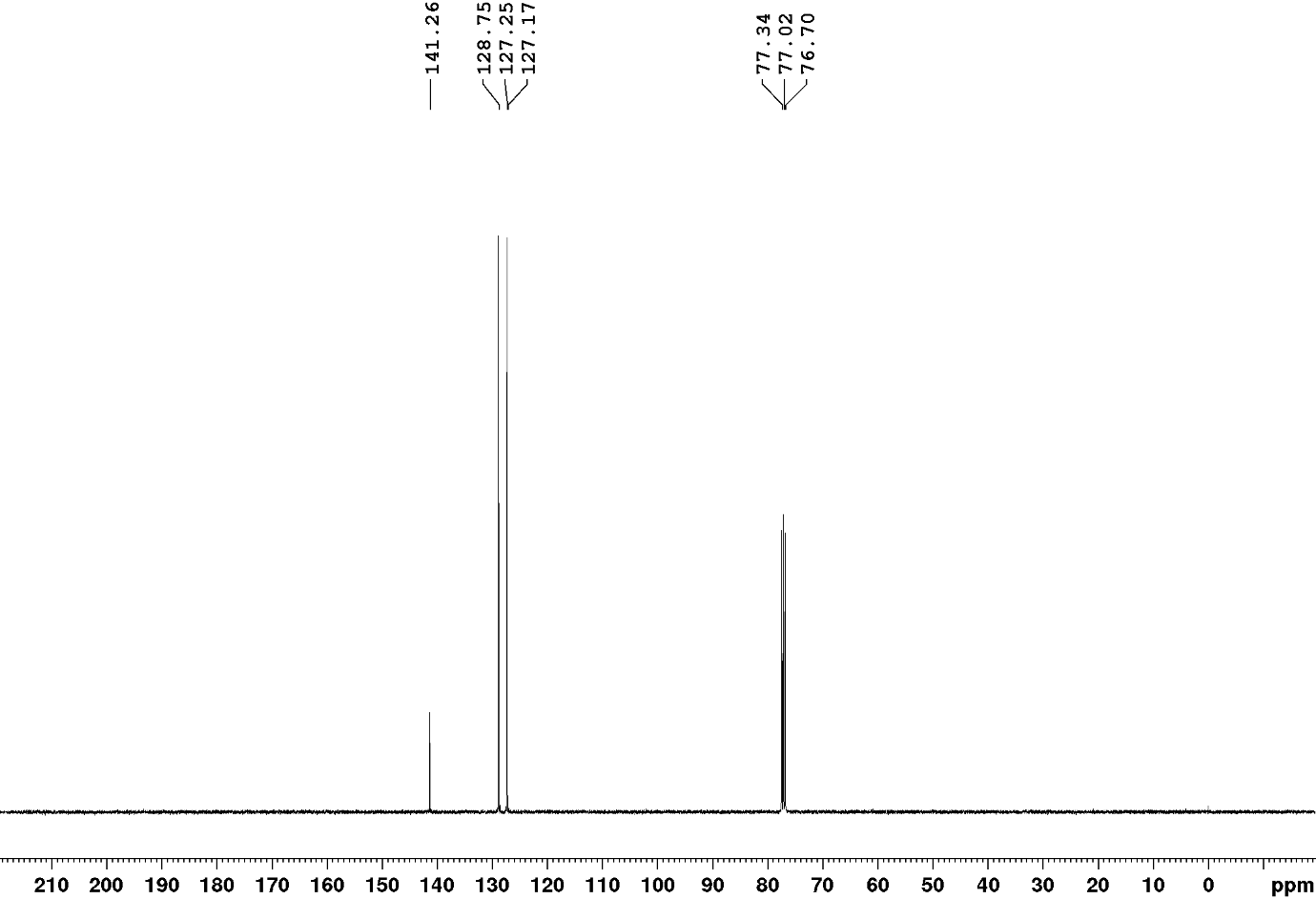

**Figure S2.** ^1^H NMR & ^13^ C NMR spectra of 4-Methoxy-1,1'-biphenyl (Table 2, Entry 4, 5 & 6)


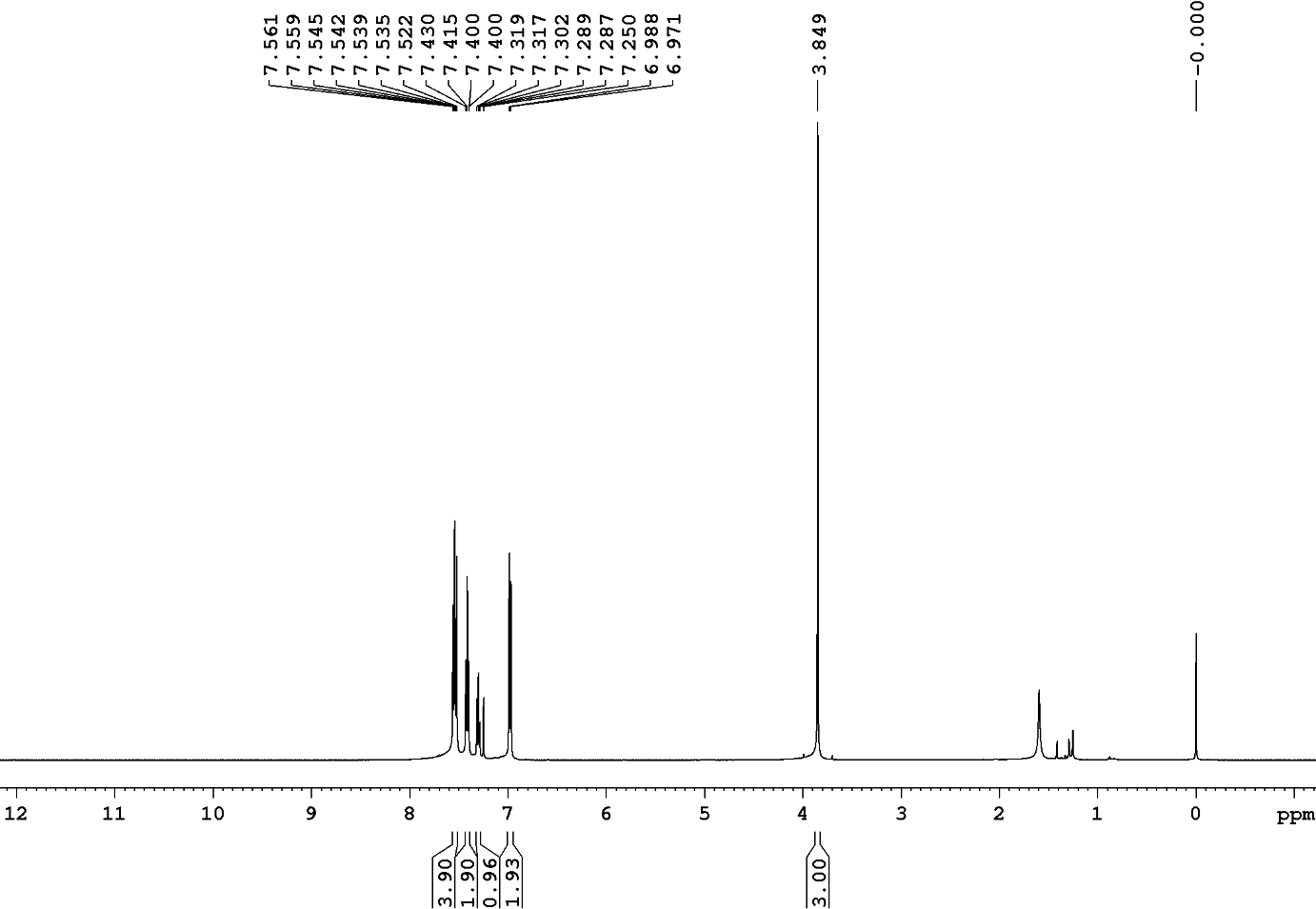

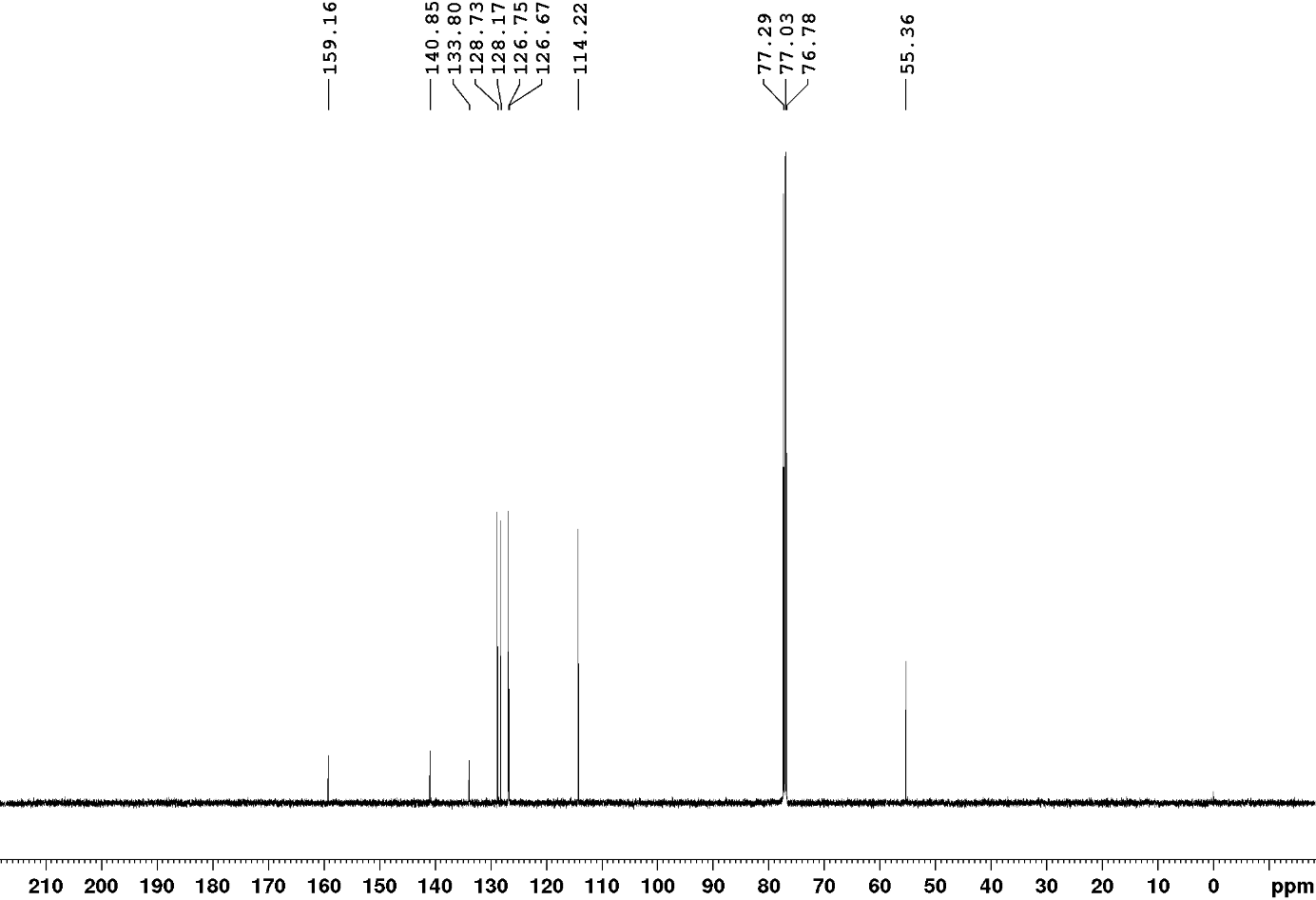

**Figure S3**. ^1^H NMR & ^13^ C NMR spectra of 1-([1,1'-biphenyl]-4-yl)ethan-1-one (Table 2, Entry 7, 8 & 9):


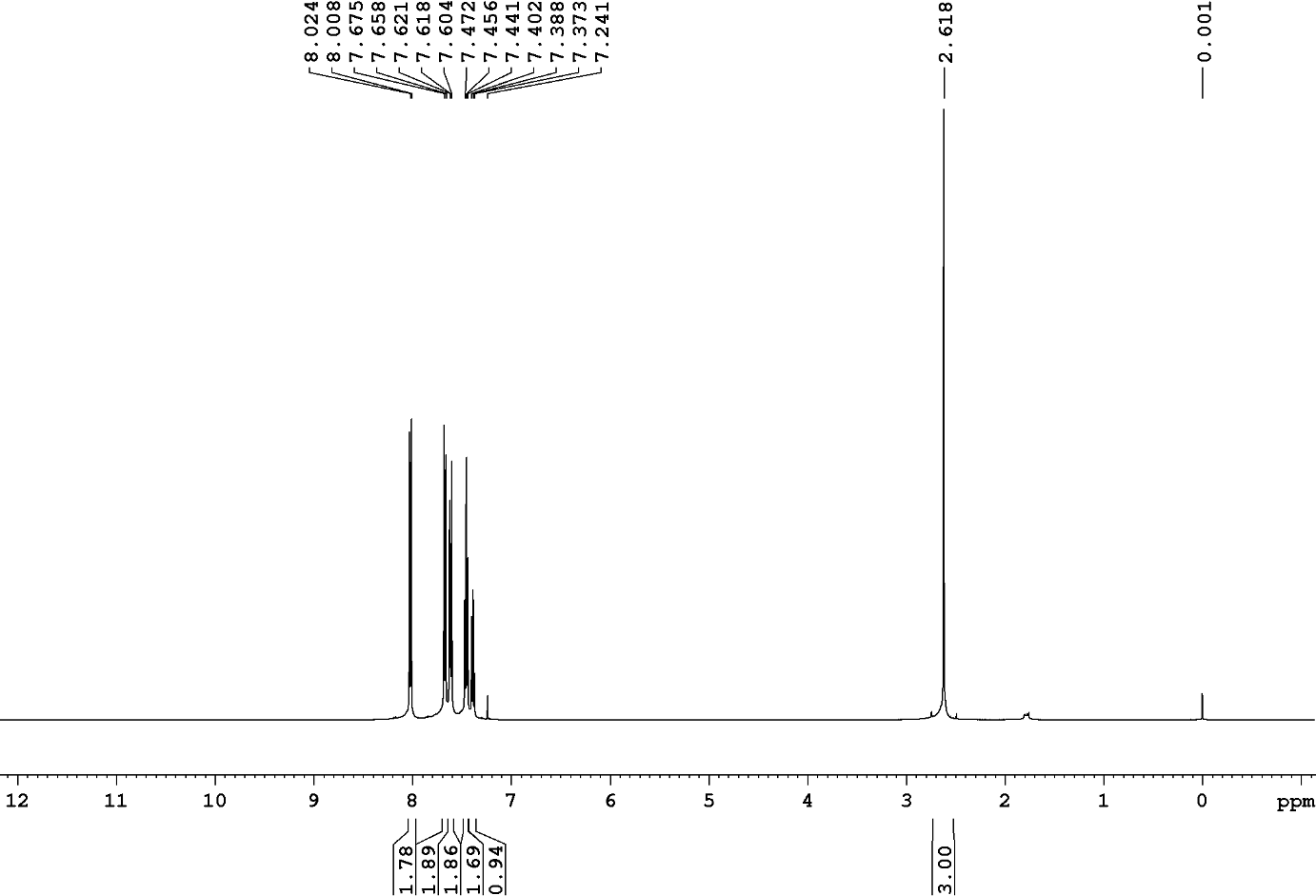

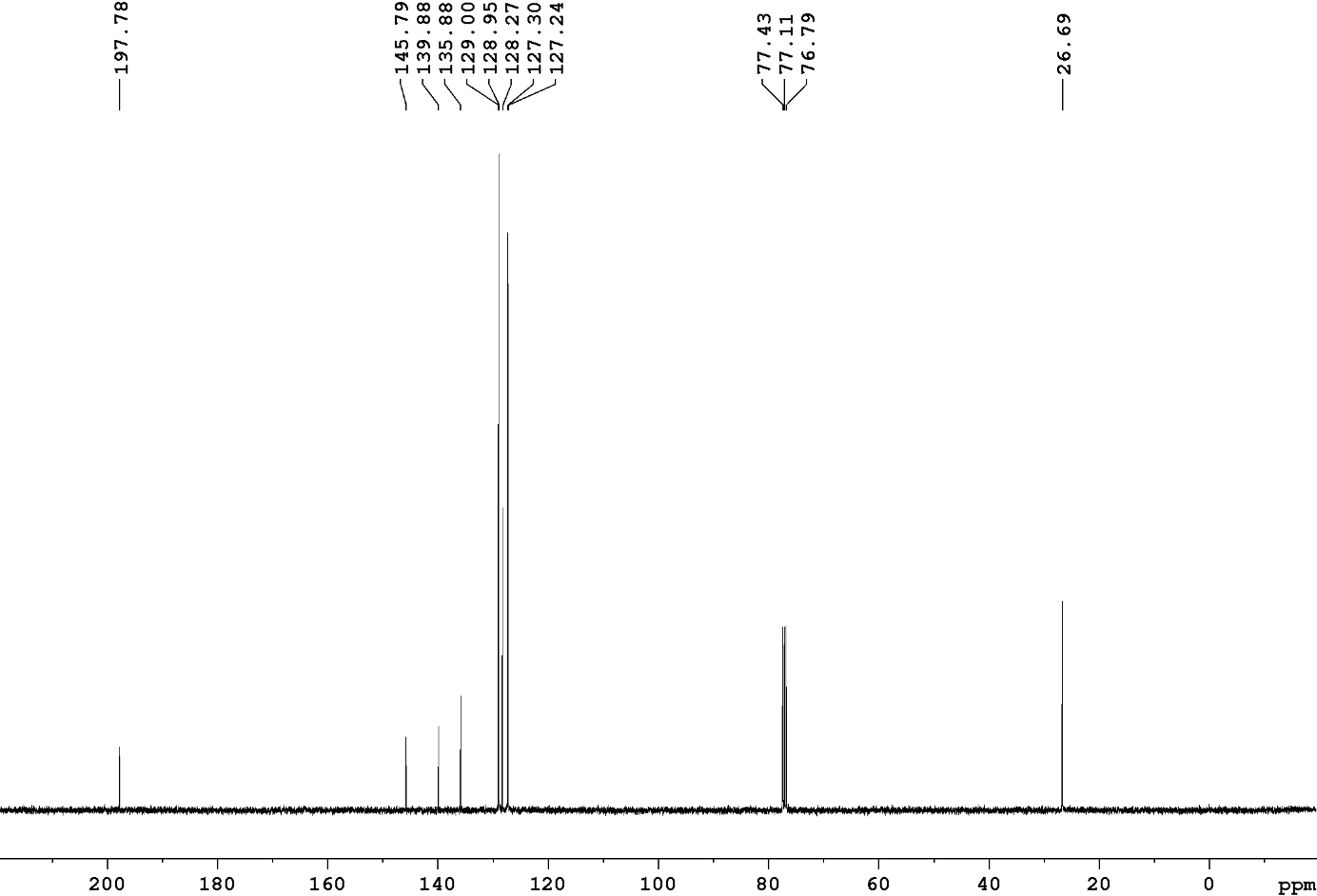

**Figure S4.** ^1^H NMR & ^13^ C NMR spectra of [1,1'-biphenyl]-4-carbaldehyde (Table 2, Entry 10)


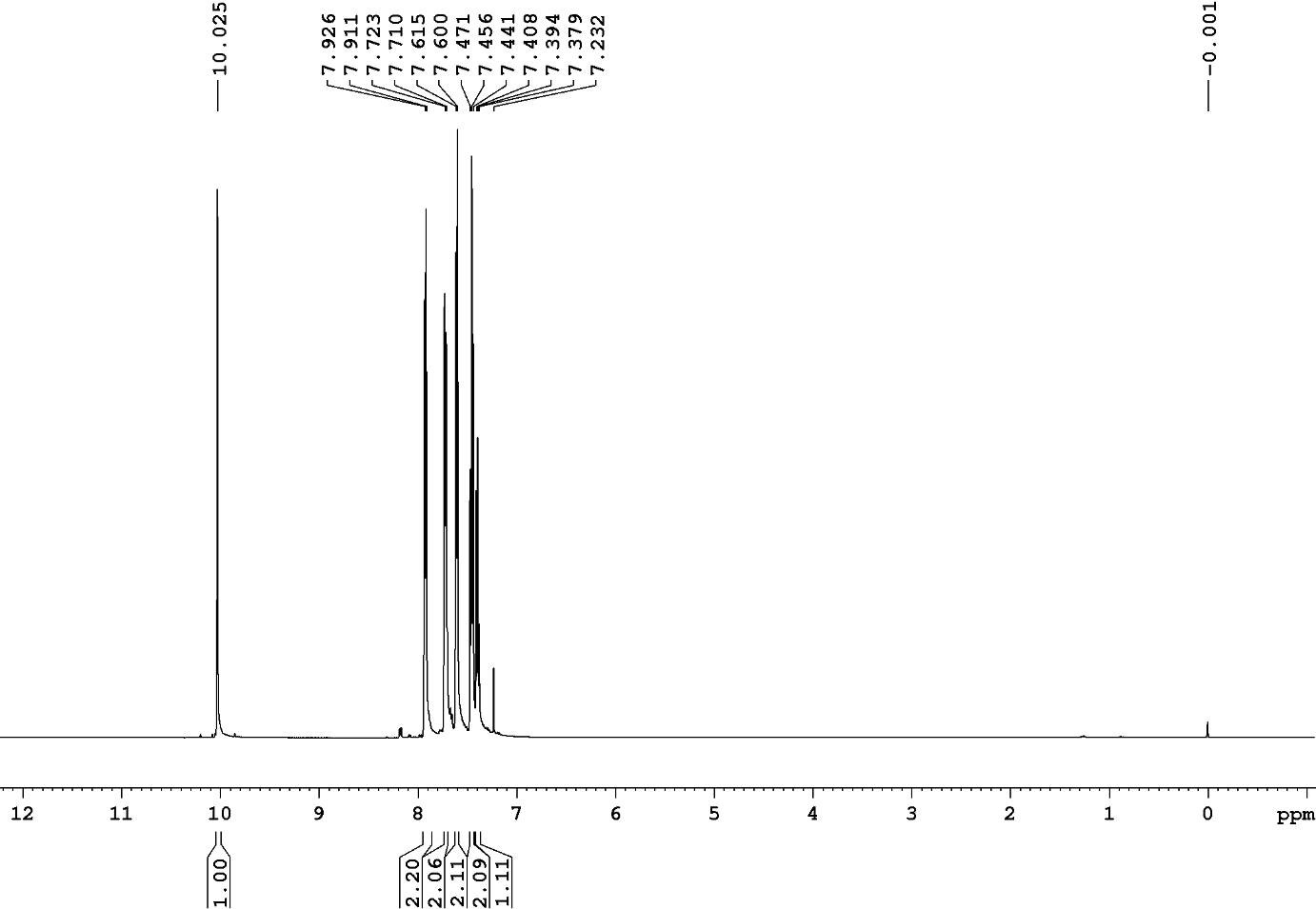

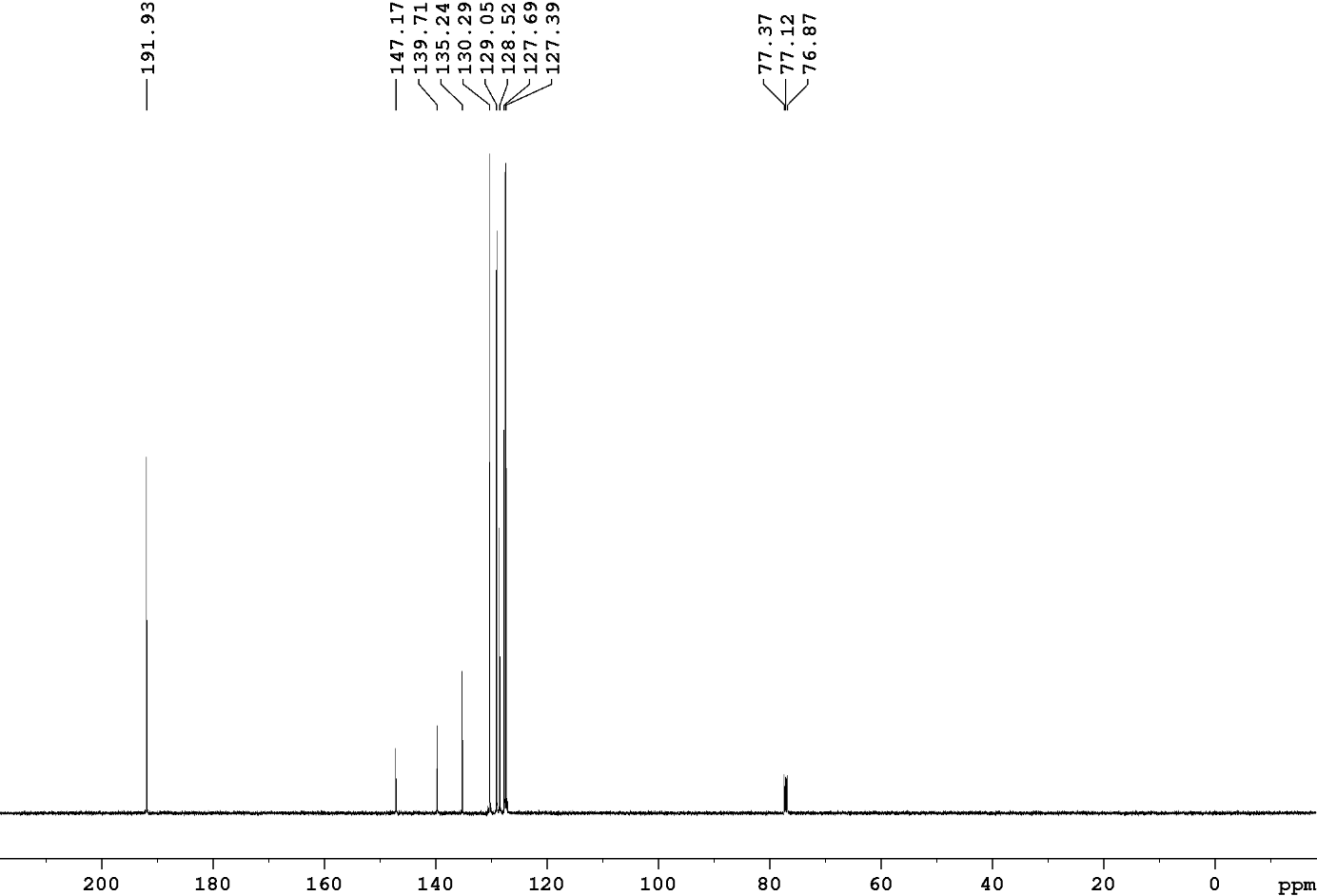

**Figure S5**. ^1^H NMR & ^13^ C NMR spectra of 2-(methoxymethyl)-1,1'-biphenyl

**Figure S6**. ^1^H NMR & ^13^ C NMR spectra of 2-nitro-1,1'-biphenyl.

**Figure S5**. TGA analysis of **TiO_2_** and **TiO_2_@BDP-PdCl_2_**. The sample was heated up to 750°C under a nitrogen atmosphere at a heating rate of 10 °C/min


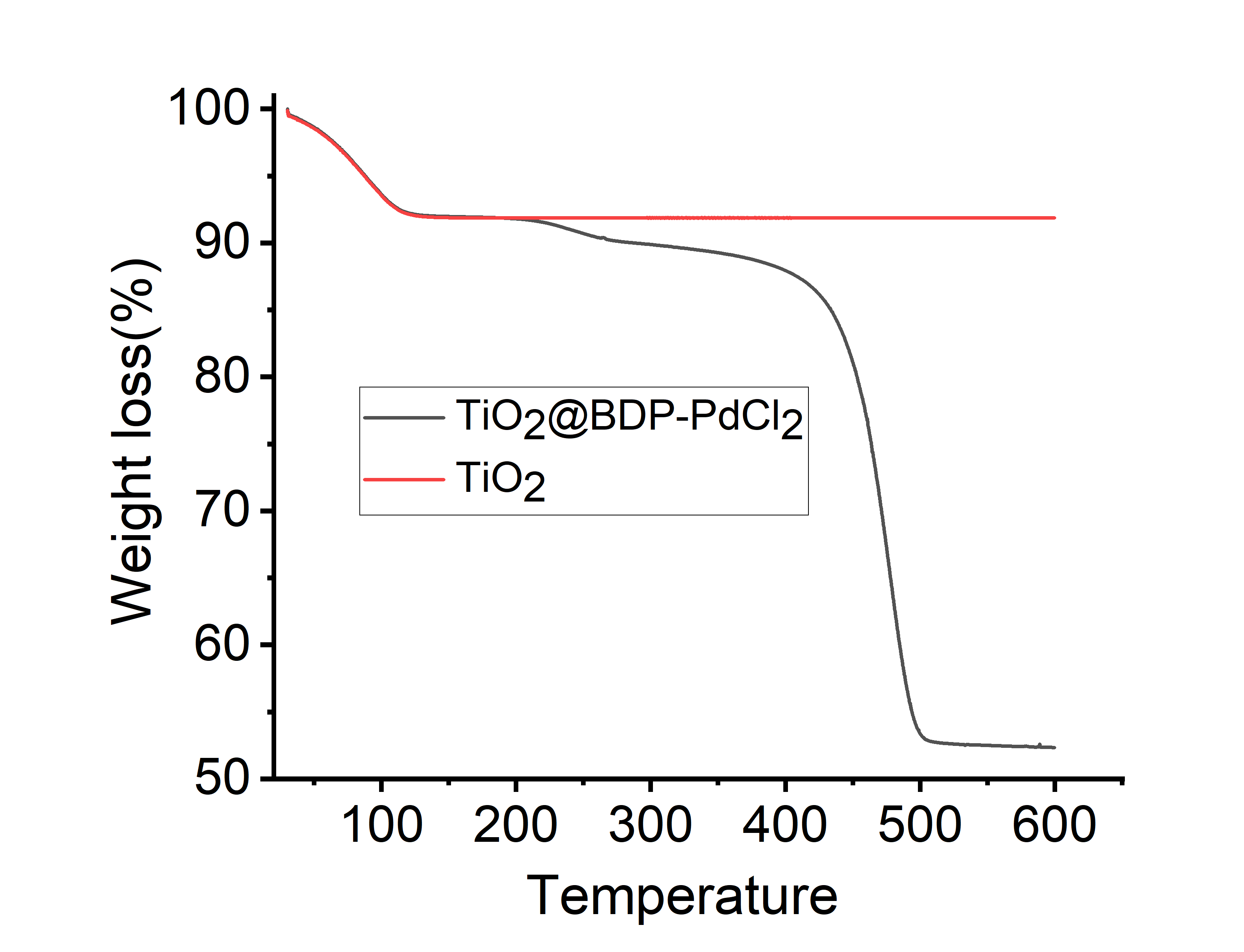


Figure S6. TEM images of TiO_2_@BDP-PdCl_2_ (a) before and (b) after five catalytic cycles revealed no noticeable changes at the end of the reactions.

Figure S7. XPS analysis of reused TiO_2_@BDP-PdCl_2_ catalyst.
